# Three weeks of heat maintenance potentiates the benefits of heat acclimation in trained females

**DOI:** 10.14814/phy2.70631

**Published:** 2025-11-18

**Authors:** Normand A. Richard, Stephen S. Cheung, Michael S. Koehle, Victoria E. Claydon, Alyssa M. Fenuta, Anita T. Coté

**Affiliations:** ^1^ Biomedical Physiology and Kinesiology Simon Fraser University Burnaby British Columbia Canada; ^2^ Richard Physiological Services Port Moody British Columbia Canada; ^3^ Department of Kinesiology Brock University St. Catharines Ontario Canada; ^4^ School of Kinesiology University of British Columbia Vancouver British Columbia Canada; ^5^ Division of Sports Medicine University of British Columbia Vancouver British Columbia Canada; ^6^ School of Human Kinetics Trinity Western University Langley British Columbia Canada

**Keywords:** extreme heat, heat adaptation, hemoglobin mass, time trial, women

## Abstract

We investigated whether heat adaptation (HA) could be maintained in trained females following an initial acclimation period. The experimental group (EXP, *n* = 11) completed 10 sessions of HA over 2 weeks, followed by nine sessions of HA maintenance (HA_M_) over 3 weeks. HA was induced with home‐based stationary cycling while overdressing. A control group (CON, *n* = 4) was exposed to heart rate‐matched thermoneutral training. Prior to and at the end of the acclimation period (PRE, MID) and following the maintenance period (POST), $\dot{V}O_{2}\max$, peak power output (PPO), and hemoglobin mass (Hb_mass_) were determined in 18°C, before a 20 km time trial (TT) in 35°C, 45% RH. During the TT, rectal and mean skin temperature (T_re_, $\bar{T}$_sk_), heart rate, peak cardiac output ($\dot{Q}\text{peak}$), and sweat rate were measured. PPO increased (*p* = 0.0003) and TT times decreased (*p* < 0.0001) from PRE to MID and POST in EXP but not CON. $\dot{V}O_{2}\max$, T_re_, $\bar{T}$_sk_, heart rate, and $\dot{Q}\text{peak}$ remained stable in both groups. Sweat rate only increased in EXP from PRE to POST (*p* = 0.0197). Hb_mass_ did not change in EXP. HA_M_ potentiated hot exercise performance compared to HA, as demonstrated by improvements in both temperate and hot conditions. While HA_M_ suffices to further develop thermal resistance, it is unsuitable to increase Hb_mass_ following 10 days of HA or 3 weeks of HA_M_. Our findings demonstrate that females can achieve HA by overdressing at home for 10 days and that HA_M_ provides further benefits.

## Graphical abstract

At‐home heat acclimation improves thermoneutral fitness and exercise performance in a hot environment in females. Heat maintenance further potentiates performance.

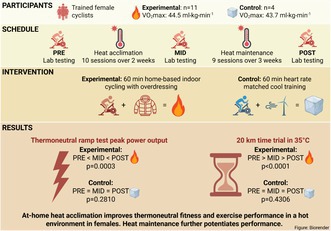

## 1 INTRODUCTION

Female participation in professional sport and arduous occupations (e.g. military, first responders), both of which can occur in hot ambient conditions, has increased in recent decades (Statistics Canada, 2024; UCI, 2025). However, females are underrepresented in thermoregulation investigations (Hutchins et al., 2021; Wickham et al., 2021) with their participation in heat research only increasing by 2% from 2016 to 2024 (Tyler et al., 2024).

Heat adaptation (HA), which incorporates artificial (acclimation) or natural heat (acclimatization), refers to collective physiological changes that improve tolerance to heat exposure. Females, like males, benefit from HA, but considerably less is known about the medium (8–14 days) to long (>15 days) HA response in females (Périard et al., 2015; Tyler et al., 2024; Wickham et al., 2021). It is undisputed that females differ from males. Females (as a group) have less muscle mass, greater fat mass, a greater ratio of surface area to mass, and smaller stature than males. Females also differ from males in their response to HA. For example, Mee et al. showed that males could achieve reductions in resting and exercise core temperature and exercise heart rate after 5 days of HA, whereas this response was only seen after 10 days in females (Mee et al., 2015). While the reader is directed to the reviews of Kelly et al. and Wickham et al. for greater details regarding sex differences in HA and female‐specific HA, exercise core temperature (T_core_), skin temperature (T_sk_), and sweat rate are improved after medium‐ and long‐term HA (Kelly et al., 2023; Wickham et al., 2021). However, less is known about performance metrics (i.e., time trial [TT]) or the cardiovascular responses (cardiac output, intravascular volumes) of females exposed to medium and long‐term HA (Kelly et al., 2023; Wickham et al., 2021).

As with fitness and training, HA will decay once the thermal stimulus is removed. HA maintenance (HA_M_) can be utilized to maintain the adaptations developed during an initial HA period by employing continued heat exposures at a lower frequency (~2–3 sessions per week) (Gibson et al., 2020; Pryor, Johnson, et al., 2019). However, HA_M_ research is currently sparse, and the limited available data are male‐centric. Additional performance enhancement has been noted with HA_M_ in males, whereby a 4 km running TT in 35°C was improved following HA and further improved after 8 weeks of twice weekly HA_M_ (Sekiguchi et al., 2022). Similarly, in males, after 10 days of HA, HA_M_ every fifth day slowed the decay in rectal temperature (T_re_), T_sk_, and heart rate when tested in the heat 25 days post HA compared to thermoneutral controls (Pryor, Pryor, et al., 2019). Lastly, once weekly HA_M_ sessions and a short re‐acclimation block appear to maintain the benefit of prior HA in elite male sailors (Casadio et al., 2016). While T_core_, heart rate, and hot exercise performance appear supported by HA_M_, little is known about the hematological responses, whether HA_M_ potentiates HA or simply maintains it, and most importantly, whether the female response emulates or differs from the male response. As such, interventions are needed to catalogue the female response to HA_M_ before we apply HA_M_ protocols to female athletes.

As elite athletes generally have maximized training‐induced hematological adaptations, they may turn to environmental stimuli such as hypoxia and heat to further enhance oxygen transport. Plasma volume (PV) expansion frequently occurs with HA (Tyler et al., 2024). It is hypothesized that the increased PV from chronic HA could lower hematocrit (HCT), thus stimulating an erythropoietic response from the renal apparatus (Donnelly, 2001; Montero & Lundby, 2018; Rønnestad et al., 2021) that would initiate the process of hemoglobin mass (Hb_mass_) elevation. Consequently increased Hb_mass_ would increase maximal oxygen consumption ($\dot{V}O_{2}\max$) (Schmidt & Prommer, 2010). This erythropoietic response has been seen with chronic HA of 25 sessions over 5 weeks (Lundby et al., 2023; Rønnestad, Hansen, et al., 2022; Rønnestad, Lid, et al., 2022). Further, it has been demonstrated that HA_M_ of three sessions per week for 3 weeks can maintain the elevated Hb_mass_ after a 5‐week HA protocol in males (Rønnestad, Urianstad, et al., 2022). Notably, of these prior studies only one trial included a female cohort (Lundby et al., 2023) and, it is unknown if chronic heat training at a lower frequency than previously reported (<5 sessions per week for 5 weeks) can increase Hb_mass_. Currently the female response to heat‐induced hematological responses remains sparsely researched. Therefore, the chief aim of this study was to measure the performance, thermoregulatory, cardiovascular and hematological responses to HA_M_ conducted following an initial HA block in trained female cyclists. As such, we hypothesized that (1) 3 weeks of HA_M_ would maintain previously acquired benefits of a 10‐session HA block and (2) that a 10‐session HA block followed by 3 weeks of HA_M_ would be sufficient to elevate Hb_mass_ in trained females.

## 2 METHODOLOGY

### 2.1 Ethical approval

Study details were provided in print, and verbal and written informed consent was provided by participants. Ethical approval was received from Trinity Western and Simon Fraser Universities (22G18, 30001393). This study conformed to the latest version of the Declaration of Helsinki and was registered in a database (ClinicalTrials.gov ID NCT06551168).

### 2.2 Study overview

**FIGURE 1:**
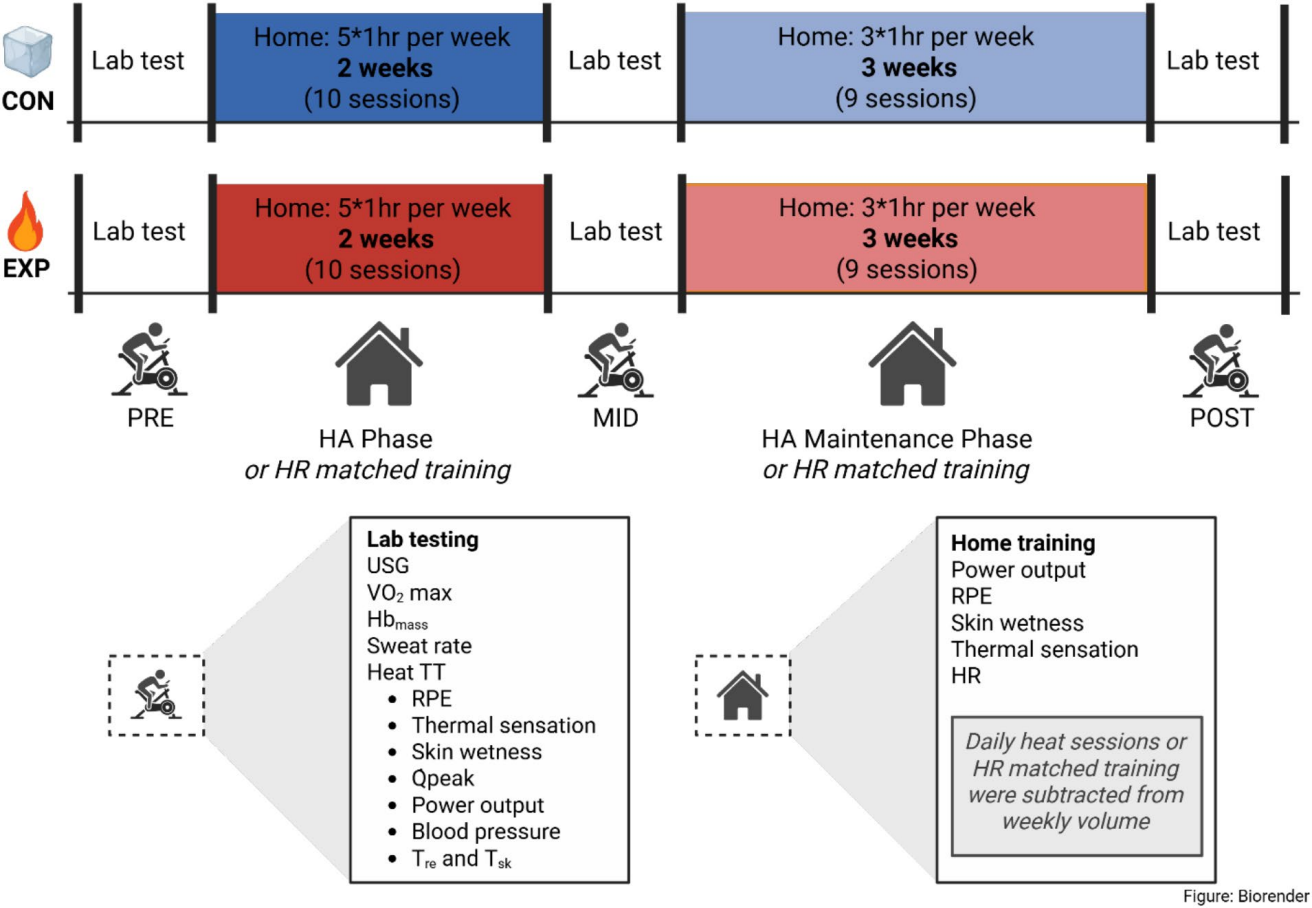
Study overview. The experimental group completed home‐based heat acclimation whereas the control group completed heart rate‐matched training. Hb_mass_, hemoglobin mass; HR, heart rate; $\dot{Q}\text{peak}$, peak cardiac output; RPE, rate of perceived exertion; $\bar{T}$_sk_, skin temperature; T_re_, rectal temperature; TT, time trial; USG, urine specific gravity; $\dot{V}O_{2}\max$, maximal rate of oxygen consumption.

Fifteen females with at least 2 years of systematic endurance training and familiarity with cycling aged 18–55 years were recruited. There was no attrition with all participants completing the study. A health screening questionnaire was administered (2024 PARQ+) and participants were instructed prior to study start to abstain from routine heat exposure and blood donation. The study was conducted in the autumn/winter of 2024‐25 in Langley, Canada (mean, min, and max temperature: 5.4°C, 1.4°C, 9.4°C, https://climate.weather.gc.ca), after the participants' race season. Laboratory visits occurred pre‐intervention (PRE), after 2 weeks of HA (MID), and after 3 weeks of HA_M_ (POST). Participants were randomized either to an experimental or control group. The experimental (EXP, *n* = 11) underwent a heat training protocol designed with the aim of developing improved TT performance in the heat and increasing Hb_mass_. A control group (CON, *n* = 4) was included to demonstrate that test habituation or participation in structured training was not the reason for the anticipated TT improvements in the HA group. The study overview can be seen in Figure 1.

### 2.3 Laboratory visits

Participants were instructed to avoid heavy exercise the day before testing and to abstain from exercise on test days. Participants were provided with standardized sports nutrition guidelines, limited their caffeine intake to habitual consumption (i.e., morning coffee), and were instructed to consume 700 mL of water in the 90 min before arriving. Immediately upon arrival to the laboratory, a urine sample was provided and analyzed for urine specific gravity (Atago, PAL‐10S, Tokyo, Japan). A euhydrated state was considered as urine specific gravity <1.020 (Casa et al., 2000).

Hb_mass_ and PV were measured with a modified version of the optimized carbon monoxide (CO) rebreathing method (Schmidt & Prommer, 2005). After 15 min of seated rest, an antecubital vein sample was collected to determine hematocrit (HCT), hemoglobin concentration ([Hb]), and COHb pre‐test values with a rapid blood gas analyzer (Prime Plus, Nova Biomedical, Waltham, USA); samples were analyzed in triplicate. If any sample differed by more than 4‐fold from the other values, the measure was assumed to be extraneous and excluded from analysis. Participants then completed the graded exercise test (GXT) and TT. After 30 min, to allow ventilation to return to baseline, a bolus of 1 mL/kg of CO (99.5% purity, Messer, Mississauga, Canada) was inhaled and held for 10 s, then rebreathed in a closed system for 110 s. An oxygen‐filled bag was attached to the system and soda lime scrubbed the CO_2_. Venous samples were taken 7 min post‐rebreathe to determine COHb post‐test. A portable CO analyzer measured the remaining CO in the lungs at 4 min post‐test and in the closed loop system (Dräger Pac6500, Lübeck, Germany). Hb_mass_ and intravascular volumes were calculated (Schmidt & Prommer, 2005). Our laboratory's % typical error (Hopkins, 2000) for venous blood Hb_mass_ is ~3%, which is inline with the % typical error of similar investigations (Carrick‐Ranson et al., 2020; Gore et al., 2006; Pethick et al., 2019).

Participants' menstrual cycle status or type of contraceptive used was recorded at each testing day. Natural cycle status was self‐reported based on the participants' personal tracking and not verified by blood hormone levels. Wearing cycling shorts and a sports bra, height was measured to the nearest cm and body mass to the nearest gram (Seca 869, Hamburg, Germany). The GXT was performed to volitional exhaustion on a cycle ergometer (Velotron, Quarq, Spearfish, USA) with exhaled gases measured with a metabolic cart (TrueOne 2400, ParvoMedics, Murray, USA) to determine $\dot{V}O_{2}\max$. The GXT was used as an indicator of fitness and performed in 18°C. The cart's pneumotach was calibrated with a 3 L syringe and the gas analyzers with a calibration gas mixture of 16% O_2_, 4% CO_2_, 80% N_2_. The GXT ramp protocol began at 50 watts with the load increasing by 0.33 watts per second (20 watts per minute). Strong verbal encouragement was provided. Peak power output (PPO) was the value achieved at test end. The GXT was considered maximal if the respiratory exchange ratio >1.1, heart rate was within 10 beats of predicted maximal heart rate, or $\dot{V}O_{2}$ plateaued despite an increasing workload. Saddle height, fore‐aft, and handlebar height were selected by the participant and ergometer setup was replicated at subsequent sessions. The gas exchange threshold and respiratory compensation point were determined using freely available online software (https://www.exercisethresholds.com) (Keir et al., 2022). The participants then rested for ~40 min. During this period, they were encouraged to drink ~250 mL of water and ingested the same standardized snack (fruit bar, FruitSource, SunRype, with 29 g carbohydrate; or energy gel, LemonSublime, GU, with 23 g carbohydrate). Participants then self‐instrumented in a private room with a single‐use rectal thermistor (Novatemp, Novamed, Elmsford, USA) inserted 15 cm past the anal sphincter. The thermistor was connected to a datalogger (LabChart, ADInstruments, Colorado Springs, USA) to record T_re_. Prior to study start, two of the single‐use thermistors were calibrated in a stable water bath against a reference thermometer (Oakton Temp360: accuracy ±0.03°C and resolution 0.01°C) between 35°C and 43°C. Further, the thermistor's resistance (Ω) was directly measured with a multimeter (Fluke 175, Everett, USA) from 33°C to 45°C as previously described (Richard et al., 2025). Body weight was measured wearing cycling shorts, a sports bra and instrumented with the thermistor, pre‐ and post‐heat TT. Water bottle weight was noted pre‐ and post‐heat TT to estimate fluid consumption. Sweat rate (L·h^−1^) was calculated as (Baker, 2017):

$$\begin{array}{c} \text{weight}\;\mathrm{pre}\;\left(\mathrm{kg}\right)-\text{weight post}\;\left(\mathrm{kg}\right)\\ \;+\text{fluid consumed}\;\left(\mathrm{kg}\right)/\text{time}\;\left(h\right)=\text{sweat rate} \end{array}$$

Participants then entered the heat chamber (35°C, RH 45%) and completed a programmed 5‐min ramp warmup (75–100 watts). Thereafter, a 20 km self‐paced TT was completed to determine exercise heat performance on the same cycle ergometer as the GXT. The goal of the TT was to determine performance changes with the intervention. Only cadence, gear ratio, and distance remaining were visible to the participants. Power data was downloaded and analyzed with opensource software (GoldenCheetah, goldencheetah.org, v3.6). Heart rate, mean skin temperature ($\bar{T}$_sk_) (Ramanathan, 1964) and T_re_ were measured continuously (Labchart, Colorado Springs, USA). $\bar{T}$_sk_ was measured on the left side with four skin temperature probes (MLT422/A, AD Instruments, Colorado Springs, USA). At km 0, 5, 10, 15, and 19, thermal sensation (Zhang et al., 2004), rating of perceived exertion (Borg 1970), and skin wetness sensation (Filingeri et al., 2015) were sought on Likert scales, and blood pressure was manually auscultated to determine systolic, diastolic and mean arterial pressure. Mean arterial pressure was calculated as:

$$\begin{array}{c} \text{diastolic arterial pressure}+1/3\times \left(\text{systolic}-\text{diastolic arterial pressure}\right)\\ \;=\text{mean arterial pressure} \end{array}$$

At km 17, peak cardiac output ($\dot{Q}\text{peak}$, L/min) was measured with inert gas rebreathing (Innocor, Cosmed, Rome, Italy). Participants were free to ingest thermoneutral water (~35°C–36°C) as they pleased. The test battery was identical at the PRE, MID and POST intervention visit.

### 2.4 Home training

The EXP group completed a HA block of five 1‐h sessions per week for 2 weeks followed by a three‐week maintenance block of three sessions per week (19 heat sessions total over 5 weeks). The heat sessions were completed at home on the participants' own smart trainer. Participants warmed up for 5 min, then cycled for 50 min at 70%–75% of maximal heart rate and 35%–45% of the PPO achieved during the GXT, while wearing a clothing ensemble intended to retain metabolic heat production while minimizing evaporation. Fan use was prohibited. The attire was composed of long underwear, wool socks, two pairs of fleece/wool pants and shirts, a rain jacket, gloves, wool hat, and an industrial size polyethylene bag with perforations for the arms and head. After 55 min, participants cooled down for 5 min and were permitted to remove the clothing ensemble. It has been shown that using a clothing ensemble in a temperate milieu is as effective as using a climatic chamber (Rønnestad, Urianstad, et al., 2022), and we have previously demonstrated in a female cohort the efficacy of overdressing in inducing moderate hyperthermia (Richard et al., 2025). Participants were instructed that for the last 15 min of the session they should feel “dripping wet” (Zhang et al., 2004) and “very hot” (Filingeri et al., 2015) and that their RPE should be no greater than 7/10. The EXP group completed a daily questionnaire to ensure that the criterion of “dripping wet” and “very hot” were met after each heat session. If necessary, additional clothing layers were added and/or if the heart rate limit permitted, power output was increased. Participants subtracted the heat training from their moderate intensity training and kept their training volume constant during the intervention period. EXP and CON uploaded their sessions to online training platforms (Strava™ or Zwift™) daily for verification by the lead researcher. As we aimed to increase Hb_mass_, both the EXP and CON group were provided with 150 mg elemental iron (FeraMAX PD, Mississauga, Canada) to be taken every other day for the study duration (Lundby et al., 2023; Stellingwerff et al., 2019) and because of anticipated sweat loss the EXP group was provide with electrolyte mix (Gatorade PERFORM, PepsiCo, Chicago, USA).

The CON group performed the same heart rate‐matched (Pethick et al., 2019) home training session as the HA group but without overdressing. The CON group was encouraged to complete their sessions outdoors. If exercising indoors they were told to wear minimal clothing, use a fan, and open windows or set their thermostat to <20°C. Participants were not blinded to which arm of the study they were assigned.

### 2.5 Statistical analysis

Data were analyzed for normality using the Shapiro–Wilk test. One‐way repeated measures ANOVA or non‐parametric tests based on normality were used to assess differences between the PRE, MID, and POST timepoints in both the EXP and CON groups. When comparing the same metric at various timepoints in the TT across PRE, MID, and POST, a two‐way repeated measures ANOVA was used. Tukey post hoc tests were conducted to identify the differences following the ANOVA. For ANOVA, we report effect size as partial eta squared interpreted as such: 0.01–0.08 small, 0.09–0.25 medium, and >0.25 as large effect size. Unpaired student's *t*‐tests were used to compare the baseline values at study entry between the EXP and CON. Data are presented as the mean and standard deviation (SD), and statistical significance was set at *p* < 0.05. Data analysis was not blinded. Sample size was determined using a power calculation to detect the anticipated 8–10% improved performance following HA (Keiser et al., 2015; Kirby et al., 2019; Lorenzo et al., 2010). Baseline power data were retrieved from a study examining the mean maximal power (MMP) profile of UCI female cyclists (Mateo‐March et al., 2022). Utilizing the 25th percentile group (representing tier 2–3 athletes), the MMP for the 30 min range (we anticipated our female cyclists would complete the 20 km TT heat performance test in ~35 min) was 236 watts. Assuming a SD of 20 watts and an 8% increase, nine females would allow us to detect a difference with an alpha of 0.05 and power of 80% (https://clincalc.com/stats/samplesize.aspx). GraphPad Prism v10.4.1 (RRID:SCR_002798) was used for data analysis and visualization.

## 3 RESULTS

All participants self‐reported as female and identified as women; characteristics are presented in Table 1. All participants completed the study with no adverse events and weight remained stable at all timepoints in both groups. The EXP group completed 19 sessions of 60 ± 0 min of heat training and the CON group completed 19 sessions of 60.9 ± 11.7 min of heart rate‐matched training (*p* = 0.0009 EXP vs. CON). Additional training volume was non‐significant between groups (EXP 81 ± 59 min, CON 89 ± 73 min per session, *p* = 0.9214). Analysis of online training records showed no participants trained more in the current study than their habitual training volume. Two participants chose to continue taking their current iron supplement; the remaining participants adhered to the provided iron routine. Because of technical difficulties one EXP resting T_re_, one EXP exercise $\bar{T}$_sk_, one EXP $\dot{Q}\text{peak}$, one CON Hb_mass_, two EXP warmup heart rate, and one EXP TT were not included in the analyses. All data mean and SD are seen in Table 2 with the table p value representing the ANOVA test significance and when significant the effect size is noted. The post hoc test *p*‐value when significant is expressed in the results text below.

**TABLE 1:** Participant characteristics.

	Age	Height	Weight	$\dot{V}O_{2}\max$		PPO	Endurance	Strength	NC	IUD Hrm	Ethnicity
	Year	cm	kg	mL/kg/min	L/min	W	h/week	Sessions/week			
EXP											
01	25	166	61.7	39.1	2.41	217	5	1	x		European
02	30	166	53.4	57.2	3.05	288	11	1		x	European and Indian
03	36	162	61.8	41.7	2.58	236	9	4	x		South American and European
04	39	161	62.7	38.8	2.43	247	5			x	East Asian (Taiwan)
05	50	161	61.5	41.1	2.53	270	5	2		x	European
06	28	167	56.8	47.7	2.71	273	10	3	x		European
07	43	164	70.2	37.0	2.60	272	5	1	x		East Asian (Chinese)
08	54	168	62.2	35.9	2.23	227	9	1	PM		European
09	41	173	59.8	48.6	2.91	292	13	3	AM		European
10	42	172	69.2	53.0	3.66	366	11			x	European
11	32	168	61.7	49.1	3.03	311	7	1		x	European
AVG	38	166	61.9	44.5	2.74	273	8.2	1.9			
CON											
21	30	159	57.4	49.6	2.85	249	10	2	x		East Asian (Chinese)
22	31	165	60.5	42.5	2.57	257	5	1	x		East Asian (Chinese)
23	23	167	61.2	48.1	2.95	291	5	1		x	European
24	52	168	82.2	34.6	2.85	274	8	1	PM		European
AVG	34	165	65.3	43.7	2.81	268	7.0	1.25			
*p* value	0.57	0.57	0.60	0.85	0.66	0.76	0.46	0.19			

*Note*: *p* values represent comparisons between groups using unpaired Student *t*‐test.

Abbreviations: AM, amenorrheic; AVG, average; CON, control; EXP, experimental; IUD Hrm, hormonal intra uterine device; NC, naturally cycling; PM, post menopause.

**TABLE 2:** Responses to acclimation protocol and HA_M_.

	EXP				CON			
	PRE	MID	POST	*p* value	PRE	MID	POST	*p* value
	AVG ± SD	AVG ± SD	AVG ± SD	Effect size^a^	AVG ± SD	AVG ± SD	AVG ± SD	Effect size^a^
Fitness data ($\dot{V}O_{2}\max$ test)								
USG	1.004 ± 0.002	1.005 ± 0.004	1.005 ± 0.003	0.8438	1.011 ± 0.006	1.009 ± 0.01	1.007 ± 0.004	0.5836
Weight (kg)	61.9 ± 4.8	61.8 ± 4.3	62 ± 4.4	0.8803	65.3 ± 11.4	65.3 ± 11.8	66.4 ± 11.5	0.2731
$\dot{V}O_{2}\max$ (L/min)	2.74 ± 0.4	2.76 ± 0.39	2.79 ± 0.39	0.1048	2.81 ± 0.16	2.88 ± 0.16	2.85 ± 0.18	0.4796
$\dot{V}O_{2}\max$ (mL/kg/min)	44.5 ± 7	44.8 ± 6.3	45.2 ± 6.6	0.2527	43.7 ± 6.8	44.9 ± 6.7	43.7 ± 6.4	0.4911
PPO (watts)	273 ± 42	281 ± 38	285 ± 39	**0.0003; 0.63**	268 ± 19	274 ± 27	276 ± 29	0.281
Watts at GET	124 ± 29	138 ± 23	150 ± 27	**<0.0001; 0.67**	134 ± 15	141 ± 25	135 ± 25	0.3772
Watts at RCP	189 ± 37	210 ± 34	214 ± 35	**<0.0001; 0.62**	196 ± 28	203 ± 23	186 ± 27	0.2568
Performance data (heat TT)								
TT time (s)	2457 ± 235	2377 ± 190	2340 ± 182	**<0.0001; 0.85**	2550 ± 109	2571 ± 107	2566 ± 129	0.4306
Resting T_re_ (°C)	37.6 ± 0.55	37.63 ± 0.32	37.81 ± 0.27	0.3955	37.58 ± 0.39	37.65 ± 0.33	37.6 ± 0.43	0.8206
Peak T_re_ (°C)	39.2 ± 0.66	39.14 ± 0.41	39.13 ± 0.6	0.8321	38.99 ± 0.51	38.83 ± 0.45	38.59 ± 0.5	0.1433
Delta T_re_ (°C)	1.6 ± 0.39	1.51 ± 0.37	1.43 ± 0.48	0.3624	1.41 ± 0.19	1.18 ± 0.2	0.99 ± 0.3	**0.0046; 1.0**
Resting $\bar{T}$_sk_ (°C)	32.57 ± 1.21	32.11 ± 0.98	31.95 ± 0.79	0.2624	31.29 ± 0.73	32.04 ± 0.96	31.72 ± 0.83	**0.0163; 0.83**
Peak $\bar{T}$_sk_ (°C)	36.35 ± 0.93	36.55 ± 0.59	36.02 ± 1.06	0.9761	36.08 ± 0.8	35.69 ± 0.28	36.26 ± 1.08	0.9306
Delta $\bar{T}$_sk_ (°C)	3.78 ± 0.91	4.44 ± 1.38	4.07 ± 1.34	0.5571	4.79 ± 0.77	3.65 ± 1.11	4.54 ± 1.1	0.1621
Sweat rate (L/h)	0.77 ± 0.17	0.87 ± 0.21	0.94 ± 0.19	**0.0197; 0.33**	0.87 ± 0.07	0.85 ± 0.1	0.89 ± 0.16	0.8075
$\dot{Q}\text{peak}$ (L/min)	15.4 ± 2.3	15.2 ± 2.3	15.1 ± 1.3	0.8821	13.8 ± 1.4	13.7 ± 1.7	14.4 ± 2.4	0.4327
End TT SAP (mmHg)	158 ± 10	164 ± 8	165 ± 9	0.1661	157 ± 4	157 ± 3	155 ± 2	0.5278
End TT DAP (mmHg)	56 ± 5	53 ± 4	49 ± 5	**0.0057; 0.51**	50 ± 3	51 ± 3	50 ± 5	0.6528
End TT MAP (mmHg)	90 ± 5	89 ± 3	88 ± 4	0.5232	85 ± 2	86 ± 2	85 ± 3	0.4068
Pre TT HR (bpm)	107 ± 14	108 ± 15	108 ± 12	0.7321	90 ± 24	91 ± 14	93 ± 14	0.8306
Average TT HR (bpm)	166 ± 9	168 ± 8	168 ± 8	0.3328	155 ± 11	151 ± 14	149 ± 17	0.1734
Average WU HR (bpm)	129 ± 13	120 ± 10	123 ± 11	**<0.0001; 0.73**	117 ± 9	120 ± 11	117 ± 6	0.2331
Pre‐TT RPE (au)	0.9 ± 0.8	1.2 ± 0.9	1.5 ± 0.7	0.9535	1.8 ± 1	1.3 ± 0.5	1 ± 1.2	0.7113
Pre‐TT Therm sens. (au)	1.5 ± 0.5	1.6 ± 0.8	1.5 ± 0.5	0.7107	1.8 ± 0.5	1.8 ± 0.5	1.3 ± 0.5	0.0731
Pre‐TT Skin wet. (au)	−0.1 ± 1.1	−0.3 ± 1.4	0.1 ± 1.4	0.1785	−0.3 ± 1	−0.5 ± 1	−0.5 ± 1	0.0731
Mid‐TT RPE (au)	5.6 ± 1.9	5.6 ± 2	6 ± 1.7	0.9535	4.8 ± 1	4.8 ± 0.5	4 ± 0	0.7113
Mid‐TT Therm sens. (au)	3.4 ± 0.7	3.5 ± 0.5	3.6 ± 0.5	0.7107	3 ± 0.8	3 ± 0.8	3 ± 0.8	0.0731
Mid‐TT Skin wet. (au)	−2.5 ± 0.5	−3 ± 0	−3 ± 0	0.1785	−3 ± 0	−3 ± 0	−2.5 ± 0.6	0.0731
End‐TT RPE (au)	8.6 ± 1.2	8.9 ± 1.3	9.4 ± 0.7	0.9535	7.8 ± 1	7.8 ± 1.7	7.8 ± 1.5	0.7113
End‐TT Therm. sens. (au)	4 ± 0	3.9 ± 0.3	4 ± 0	0.7107	3.8 ± 0.5	3.5 ± 1	3.8 ± 0.5	0.0731
End‐TT Skin wet. (au)	−3 ± 0	−3 ± 0	−3 ± 0	0.1785	−3 ± 0	−3 ± 0	−3 ± 0	0.0731
Hematological data								
Hb_mass_ (g)	645 ± 100	679 ± 114	633 ± 93	0.3019	694 ± 129	618 ± 81	628 ± 70	0.1993
PV (mL)	3017 ± 547	3098 ± 460	2959 ± 430	0.5542	3277 ± 502	2895 ± 492	2987 ± 391	0.1638
BV (mL)	4940 ± 819	5098 ± 745	4834 ± 686	0.4389	5370 ± 862	4747 ± 736	5040 ± 684	0.3191
RBCV (mL)	1923 ± 306	2000 ± 35	1875 ± 269	0.3494	2093 ± 389	1852 ± 251	2000 ± 350	0.5425
HCT (%)	43 ± 3	43 ± 2	43 ± 2	0.7435	43 ± 2	43 ± 2	43 ± 2	0.9602
[Hb] (g/dL)	14.4 ± 1.1	14.5 ± 0.8	14.5 ± 0.7	0.8254	14.2 ± 0.8	14.4 ± 0.6	14.2 ± 0.7	0.7594

*Note*: Data are presented as mean ± standard deviation. *p* values represent comparisons within groups using one‐way repeated measures ANOVA, and two‐way repeated ANOVA for RPE, thermal sensation and skin wetness. Bold indicates *p* < 0.05.

Abbreviations: $\bar{T}$_sk_, skin temperature; $\dot{V}O_{2}\max$, maximal rate of oxygen consumption; $\dot{Q}\text{peak}$, peak cardiac output; [Hb], hemoglobin concentration; AU, arbitrary units; BV, blood volume; DAP, diastolic arterial pressure; GET, gas exchange threshold; Hb_mass_, hemoglobin mass; HCT, hematocrit; HR, heart rate; MAP, mean arterial pressure; PPO, peak power output; PV, plasma volume; RBCV, red blood cell volume; RCP, respiratory compensation point; RPE, rate of perceived exertion; SAP, systolic arterial pressure; Skin wet, skin wetness; Therm sens, thermal sensation; T_re_, rectal temperature; TT, time trial; USG, urine specific gravity; WU, warmup.

^a^ Effect size only shown for significant *p* values.

### 3.1 Physiological response to HA and HA_M_

**FIGURE 2:**
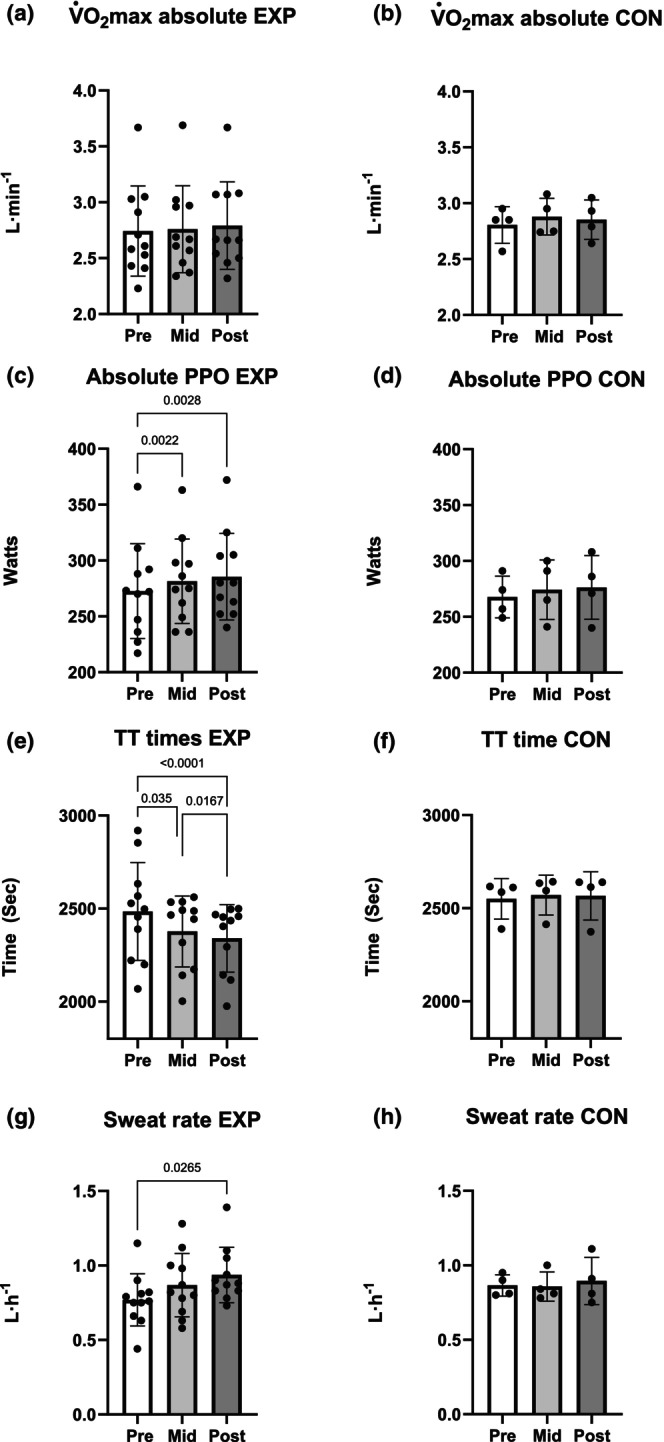
Performance variables. (a, b) $\dot{V}O_{2}\max$, (c, d) PPO, (e, f) TT, and (g, h) sweat rate in the EXP and CON groups. CON, control group; EXP, experimental group; PPO, peak power output; TT, time trial; $\dot{V}O_{2}\max$, maximal rate of oxygen consumption.

Rest, peak and delta T_re_ and $\bar{T}$_sk_ between the three timepoints were not different (Table 2) in EXP. In CON, resting $\bar{T}$_sk_ increased from PRE to POST and PRE to MID (*p* = 0.0252, *p* = 0.0337). In CON delta T_re_ was smaller in POST than PRE (*p* = 0.0140), while resting T_re_ was not different between the three timepoints, however peak T_re_ was lower in MID versus POST (*p* = 0.0307) (Table 2). Sweat rate increased by 18% from PRE to POST (*p* = 0.0265) in EXP but not in CON (Table 2, Figure 2g,h). When comparing perceived metrics at km 0, 10 and 20 between the three time points, skin wetness was significantly increased between PRE versus MID and PRE versus POST at km 10 (*p* = 0.0394, p = 0.0394) in EXP. RPE and thermal sensation did not differ in EXP or CON. In EXP, average heart rate over the standardized 5‐min warm‐up was significantly higher in PRE versus MID and PRE versus POST (*p* = 0.0005, *p* = 0.0128).

$\dot{Q}\text{peak}$, mean arterial pressure, systolic arterial pressure, resting heart rate inside the heat chamber, and average TT heart rate did not differ between timepoints in either condition, however diastolic arterial pressure at km 19 of the TT was significantly lower at POST versus PRE (*p* = 0.0036) in EXP (Table 2). Absolute and relative values for Hb_mass_, BV, PV, and red blood cell volume were not significantly different between timepoints in EXP (Table 2). In CON, absolute Hb_mass_ and BV decreased from PRE to MID (*p* = 0.0358, *p* = 0.0263).

### 3.2 Fitness response

**FIGURE 3:**
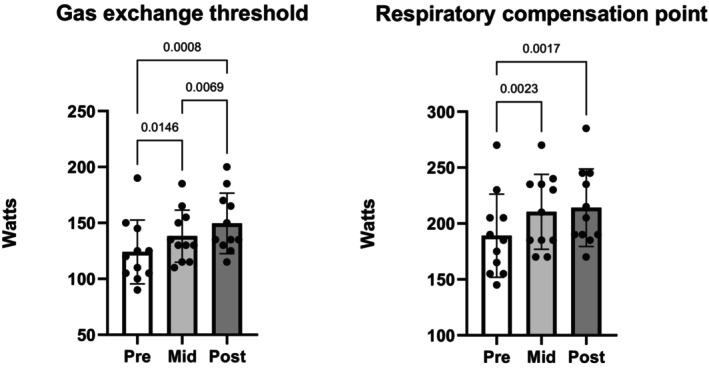
Gas exchange threshold and respiratory compensation point in the experimental group. Control group data are all non‐significant and can be viewed in Table 2.

Absolute and relative $\dot{V}O_{2}\max$ did not differ at any timepoint in either group, whereas absolute PPO increased 3.1% from PRE to MID (*p* = 0.0022) and by 4.5% PRE to POST (*p* = 0.0028) in EXP but not in CON (Table 2, Figure 2a–d). In EXP, the power output at the gas exchange threshold increased from PRE versus MID by 10% (*p* = 0.0146), PRE versus POST by 17% (p = 0.0008), and MID versus POST by 7.6% (*p* = 0.0069) and the respiratory compensation point increased from PRE versus MID by 10.2% (*p* = 0.0023) and PRE versus POST by 11.7% (*p* = 0.0017) (Figure 3, Table 2). Neither the gas exchange threshold nor respiratory threshold changed in CON (Table 2).

### 3.3 Performance response

**FIGURE 4:**
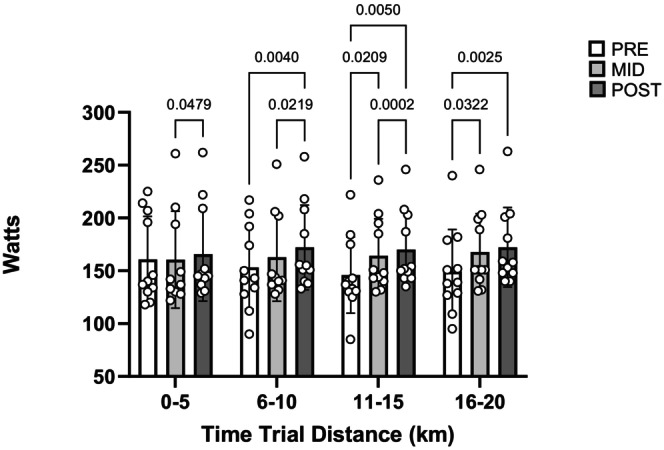
Heat time trial power output by epochs in the experimental group. Control group data are all non‐significant (data not shown).

TT times were significantly lower PRE to POST (*p* < 0.0001), PRE to MID (p = 0.035), and MID to POST (*p* = 0.0167) in EXP and did not change in CON (Table 2, Figure 2e,f). TT average power was significantly increased from PRE to POST (*p* = 0.0002), MID to POST (*p* = 0.0231), and was approaching significance from PRE to MID (*p* = 0.05) in EXP. When divided into four 5‐km epochs, analysis of the TT demonstrated a significant effect for timepoint (*p* < 0.0001) for EXP but not for CON. Within‐timepoints power output by epoch was non‐significant for both groups. However, when examining epochs between timepoints, the PRE versus MID TT had significantly greater average power for km 11–15 and km 16–20 (*p* = 0.0209, *p* = 0.0322), the PRE versus POST TT had significantly greater power at km 6–10, 11–15, 16–20 (*p* = 0.0040, *p* = 0.0050, *p* = 0.0025), whereas the MID versus POST TT had significantly higher power at km 0–5, 6–10, and 11–15 (*p* = 0.0479, *p* = 0.0219, *p* = 0.0002; Figure 4). Power output between epochs did not differ across timepoints in CON.

## 4 DISCUSSION

In a heterogenous sample of 11 trained females, we observed increased PPO and reduced TT time following both HA and HA_M_. HA_M_ not only maintained the physiological changes necessary to defend exercise capacity in the heat but potentiated performance. Conversely, in our control group, both fitness and performance remained stable.

### 4.1 Response to 10 days of HA

Data examining females' response to medium‐term HA is sparse, with the female sweat rate response being slightly contradictory. As in our trial, Kirby et al. showed no change in sweat rate at 9 days post HA in females (Kirby et al., 2019). Similarly, O'Toole et al. also showed no change in sweat rate following 10 days HA in females (O'Toole et al., 1983), and this was echoed by Stephens and Hoag, who also failed to observe a change in sweat rate using either dry or humid heat in females (Stephens & Hoag, 1981). Conversely, some have shown that females (but not males) increased sweat rate after either short‐term HA and/or medium‐term HA (Horstman & Christensen, 1982; Mee et al., 2015). While fitness status was not strikingly different from the above studies, the protocol for HA and how sweat rate was quantified (i.e., during the HA session vs. during a performance test) varied considerably and likely accounts for discrepancies. Lowered resting T_re_ is a hallmark of HA (Périard et al., 2015). However, we are not surprised that resting T_re_ did not change between timepoints. To minimize laboratory visits, the GXT was conducted 40 min prior to the TT. As such, metabolic heat production from the GXT likely masked any resting T_re_ differences. Conversely, we attribute the absence of peak T_re_ difference to increased metabolic heat production in the MID and POST trial as power output was greater than PRE. During our fixed‐rate warm‐up, we observed a decreased heart rate in MID and POST versus PRE, which we interpret as a sign of heat adaptation, thus supporting the effectiveness of our intervention.

Similar to work in males and mixed‐sex cohorts, we show no increase in $\dot{Q}\text{peak}$ following HA (Horstman & Christensen, 1982; Keiser et al., 2015; Sotiridis et al., 2019). The most logical explanation for the absence of enhancement in $\dot{Q}\text{peak}$ is the lack of increase in PV. In addition, our $\dot{V}O_{2}\max$ values, measured in a thermoneutral room, also remained stable. In studies that do show an increased $\dot{Q}\text{peak}$, there was also an increase in $\dot{V}O_{2}\max$ and in PV following HA (Lorenzo et al., 2010). In Horstman & Christensen, the lack of change in $\dot{Q}\text{peak}$ was attributed to their participants' fitness (females $\dot{V}O_{2}\max$ of ~47 mL/kg/min), which could be the case in our cyclists (Horstman & Christensen, 1982). Finally, barring dehydration (>3% body mass) some suggest that $\dot{Q}$ remains relatively stable during resting and prolonged hyperthermic exercise (Travers et al., 2020).

Following medium‐term HA, our participants demonstrated increased PPO in the GXT and a rightward shift in the gas exchange threshold and respiratory compensation point despite no change in either absolute or relative $\dot{V}O_{2}\max$. The current heat training literature is mixed regarding improvements in temperate $\dot{V}O_{2}\max$ and PPO following HA. Lorenzo and colleagues, in a mostly male trial, showed improvement after acclimation, while others have shown no improvements after acclimation or acclimatization in male participants (Karlsen et al., 2015; Keiser et al., 2015; Lorenzo et al., 2010). Our GXT was performed 48 h after the last heat training session, and it has been suggested that 96 h is the optimal time for maximizing $\dot{V}O_{2}\max$ and PPO following 10 days of HA (Waldron et al., 2019). While scientifically best practice, from an applied sports science setting it's unlikely endurance athletes would abstain from exercise for 4 days. Our observations may reflect that medium‐term HA can improve exercise metabolism. Sawka et al., have shown that following 10 days of HA, metabolic rate during a standardized test in both cool and hot conditions was 5% and 3% lower than pre HA (Sawka et al., 1983). Further, in females exposed to 2 h of daily steady‐state work in HA for 10 days, the $\dot{V}O_{2}$ needed for the 2‐h sessions decreased from 39% to 36% despite no change in $\dot{V}O_{2}\max$ pre and post intervention (O'Toole et al., 1983). As power output at the gas exchange threshold and respiratory compensation point increased in our EXP group, we suggest that the increased PPO despite no change $\dot{V}O_{2}\max$ resulted from enhanced metabolism. This may also explain the responses in other HA studies that showed increased PPO following HA but with no clear mechanistic explanations (Neal et al., 2016; Rendell et al., 2017; Sotiridis et al., 2019). Future research exploring the ergogenic effects of HA should continue exploring the effects of enhanced metabolism and efficiency following HA.

Our finding of improved performance is supported by Kirby et al., who administered a 15‐min self‐paced TT in 35°C and 30% RH prior to, and following 4 and 9 days of HA (Kirby et al., 2019). While 4 days of HA was insufficient to improve performance, 9 days of HA increased performance, as in the present study. While our data of increased performance following medium‐term HA is similar to the male response (Keiser et al., 2015; Lorenzo et al., 2010), we contribute to the limited female literature by showing that medium‐term HA improves self‐paced exercise. When examining the TT over time, it becomes evident that HA and HA_M_ permitted higher work capacity at later segments. Of particular interest is the significant increase in power in the third epoch following HA and HA_M_. In pacing prolonged efforts, negative splits are considered ideal as they allow an increase in pace where the consequences of “blowing up” are less catastrophic, while limiting the rise in metabolites and lowering the rate of carbohydrate depletion (Abbiss & Laursen, 2008). In PRE the fastest quarter was km 0–5, resulting in a positive pacing profile. MID and POST did not show true negative splits but rather an even pacing strategy with the first quarter being the slowest, but with power output in the first quarter still being higher than PRE, and the remaining three quarters maintaining the work rate until task end. For both the GXT and TT a training or habituation effect is unlikely. First, participants' previous training volume did not increase during the study and all participants were familiar with cycling. Secondly both absolute and relative $\dot{V}O_{2}\max$ remained stable. In addition, no change in fitness or performance occurred in our control group. Lastly, it is unlikely that a learning effect contributed to the increased TT performance. The improved power only occurred in EXP, and within the EXP group, the average TT and, as seen in Figure 4, individual epoch power outputs increased in MID and POST.

### 4.2 Response to HA_M_

Our intervention supports our first hypothesis and demonstrates that HA_M_ not only preserves the benefits of HA but further improves fitness and performance in females. Evaporation of sweat alongside peripheral vasculature vasodilation is the primary mechanism by which hyperthermia is avoided in humans (Périard et al., 2015). Significantly greater sweat rate at POST demonstrates that our intervention was effective in improving sudomotor function with long‐term HA. Regarding perceived metrics, skin wetness was significantly greater at the 10 km mark in MID and POST versus PRE, as reflected by the greater sweat rate in the POST trial.

The PPO at $\dot{V}O_{2}\max$ remained elevated above PRE at the POST timepoint alongside the increased gas exchange thresholds and respiratory compensation point. In similar trials, Lundby et al., showed improved power output at 3 mmol [La], and increased 15 min peak power after 5 weeks of heat training using 5 sessions per week in a cohort of female and male cyclists. Likewise Cubel et al., showed improved PPO after 5 weeks of heat training using six sessions per week in male cyclist (Cubel et al., 2024; Lundby et al., 2023). Comparing our data to the two aforementioned studies it appears that reduced frequency (HA_M_) can maintain fitness advantages developed with HA.

In our study, HA_M_ further improved TT performance. A meta‐analysis indicated performance outcomes following HA remained elevated for 1–2 weeks following heat exposure cessation (Daanen et al., 2018). With physical training, an overload period followed by rest can lead to supercompensation. Similarly, an enhanced thermal response may occur following a brief removal from HA. In one study examining this effect, males undertook a 9‐day HA protocol, followed by 3 days of severe heat exposure with measurements taken during a decay period at 3, 7, and 18 days post HA. T_re_ and heart rate at rest and after the 60 min of exercise were lower in the decay period than after the HA period (Daanen et al., 2011). Furthermore, time‐to‐exhaustion increased significantly in the decay period versus at the start or end of their HA block. Although we did not use a decay period, our reduced frequency of overdressing‐induced hyperthermia could have permitted the undertaking of thermoregulatory adaptations. Length of decay, as indicated above, seems critical. Cubel tested the response of a two‐week decay period (no heat, simply habitual training) and showed decreased time‐to‐exhaustion in the heat following 5 weeks of heat training (Cubel et al., 2024). When comparing our performance data to heat training studies using a 5‐day per week for 5 weeks model, performance response varies. For example, in elite cross‐country skiers, no change in a 15‐min running TT occurred following heat training (Rønnestad, Lid, et al., 2022), and in elite cyclists, no improvement as compared to a control group occurred in a 15‐min TT after heat training (Rønnestad et al., 2021). Conversely, in a pooled cohort of elite male and female cyclists, 15‐min TT performance is increased after heat training (Lundby et al., 2023) and using a weighted performance index (power at 4 mmol·L^−1^ [La], PPO, and 15 min TT power), heat training improved performance versus control training in male cyclists (Rønnestad, Urianstad, et al., 2022). From the above, if exercise performance is the key variable of interest, it appears that HA and allowing a decay maintain performance. Using a “5 × 5” model may or may not improve performance, whereas using an initial HA block followed by HA_M_ clearly improves performance in females.

### 4.3 Hematological response to HA and HA_M_

Contrary to our second hypothesis, our intervention did not increase Hb_mass_ as previously reported following 5 or 3 weeks of heat training (Cubel et al., 2024; Lundby et al., 2023; Oberholzer et al., 2019; Rønnestad, Lid, et al., 2022; Rønnestad, Urianstad, et al., 2022) or following HA_M_ (3 sessions/week for 3 weeks) after 5 weeks of HA (Rønnestad, Urianstad, et al., 2022). Our participants took 150 mg of iron every other day during the intervention, but perhaps it was insufficient. When Hb_mass_ was increased in a cohort of females and males, participants took 100 mg of iron 2 weeks prior to and for the study duration (Lundby et al., 2023). While none of our participants reported being anemic, perhaps some began the intervention with suboptimal iron status, and our supplementation approach was insufficient to fully address this. Lastly, it has been suggested that menstrual cycle fluid shifts could cloud changes in PV (Kelly et al., 2023). We do not believe this was the case in our participants, as previous investigations have shown that PV changes across the follicular and luteal phase are non‐significant (Aguree et al., 2020). We hypothesized that an initial 10‐day HA block would expand PV, and that the HA_M_ phase would maintain the expansion and promote erythropoiesis. Our results do not support this, as HA_M_ was not sufficient to drive hematological adaptations after 10 days of HA. In all, it appears that a “5 × 5” model is necessary to increase Hb_mass_, as our study and other shorter HA interventions do not increase Hb_mass_ (Nielsen et al., 1993; O'Toole et al., 1983; Patterson et al., 2004; Racinais et al., 2024).

### 4.4 Home‐based overdressing

Active HA generally occurs in an environmental chamber or a hot climate, both of which can be cost or logistically prohibitive. Overdressing to induce hyperthermia, while not a new concept, has been sparsely used in HA research. Conversely, much work has been done to negate the effects of attire which induces hyperthermia (McLellan et al., 2013). In males, overdressing when cycling outdoors in a temperate climate can increase T_core_ compared to cycling in regular attire as measured by ingestible thermometer capsules (Stevens et al., 2017). Yet using overdressing with outdoor cycling to induce HA is ineffective (Stevens et al., 2018). Knowing T_core_ during home‐based overstressing would be ideal but is not crucial. A previous study using indoor home‐based cycling with overdressing effectively induced HA in male cyclists (Cubel et al., 2024), and this study did so in female cyclists. Both studies used perceptual cues and physiological anchors but without T_core_ monitoring. We considered using wearable devices which estimate T_core_ by means of biometrics and predictive algorithms; however, they proved to be inaccurate while RPE and thermal sensation seemed more reliable (Richard et al., 2025). Our data provide athletes, coaches, and sports scientists with a low‐tech, cost‐friendly, and effective alternative to traditional HA methods.

### 4.5 Considerations and limitations

Our heterogenous sample represents the active female population as a whole and increases external validity. However, it is possible that, for some measures, a more homogenous sample may have produced different results. Ultimately the performance output metrics increased significantly, demonstrating that our intervention was effective. Although we report on menstrual cycle status, for practical reasons no attempts were made to control test time according to menstrual cycle phase so that participants could start the intervention when most feasible for them. We do not expect this impacted our results as we previously showed that physiological and perceptual responses do not differ in response to exercise during thermal stress with repeated testing in luteal and follicular phases (Richard et al., 2025). In addition, self‐paced exercise performance has been shown to be unaffected under hot environments across the menstrual cycle in well‐trained ($\dot{V}O_{2}\max$~57 mL·kg·min^−1^) women (Lei et al., 2017). As the intervention occurred at home, we cannot state the magnitude and length of the increase in T_re_ during the heat training. However, we have previously shown the efficacy of our protocol in inducing moderate hyperthermia (Richard et al., 2025), and our methods align with other home‐based heat training studies (Cubel et al., 2024). As such a trade‐off of this home‐based intervention is the lack of objective heat load quantification. Sweat rate was not calculated nude, therefore a slight error may have been introduced. However, as participants wore their same bib shorts and sports bra for all trials, sweat trapped in clothing would be consistent within individuals. Lastly, we tested fewer participants in our control group (*n* = 4) than our experimental group (*n* = 11). As the main goal of our control group was to ensure that training per se or test habituation did not account for the anticipated decrease in hyperthermic TT times, our objective was met. However, we encourage future female thermoregulatory studies to evenly match their control and experimental group and to further explore HA_M_ protocols with two sessions per week and/or of longer durations.

## 5 CONCLUSIONS

Increased rates of female participation in sports and arduous occupations in hot environments require an advanced understanding of the female response to HA. Our protocol significantly improved thermoneutral fitness and exercise performance in a hot environment. Further, we show that thrice weekly HA_M_ not only maintains the benefits of HA but potentiates performance. Further work is needed to confirm or refute our observed lack of hematological changes.

## AUTHOR CONTRIBUTIONS

**Normand A. Richard**: Conceptualization, data collection, data curation, formal analysis, investigation, writing—original draft. **Stephen S. Cheung**: formal analysis, subject matter expertise, writing—draft review. **Michael S. Koehle**: formal analysis, writing–draft review. **Victoria E. Claydon**: formal analysis, writing–draft review. **Alyssa M. Fenuta**: data collection, writing—draft review. **Anita T. Coté**: data curation, formal analysis, investigation, writing—draft review.

## Data Availability

Data relevant to the study are included in the article. Raw data can be made available upon reasonable request.
